# General Practitioners Views of Implementing a Stratified Treatment Approach for Low Back Pain in Germany: A Qualitative Study

**DOI:** 10.1371/journal.pone.0136119

**Published:** 2015-08-31

**Authors:** Sven Karstens, Stefanie Joos, Jonathan C. Hill, Katja Krug, Joachim Szecsenyi, Jost Steinhäuser

**Affiliations:** 1 Department of General Practice and Health Services Research, University Hospital Heidelberg, Heidelberg, Germany; 2 Institute of Primary Care and Health Sciences, Keele University, Keele/Stoke-on-Trent, United Kingdom; 3 Institute of Family medicine, University Hospital Schleswig-Holstein Campus Lübeck, Lübeck, Germany; Toronto Western Hospital, CANADA

## Abstract

**Background and Objective:**

The STarT Back stratified primary care approach has demonstrated clinical and cost effectiveness in the UK, and is commonly used by General Practitioners (GPs). However, it remains unknown how this approach could be implemented into the German healthcare system. The aim of this study was therefore to explore the views and perceptions of German GPs in respect to using a stratified primary care for low back pain (LBP).

**Methods:**

A 90-minute think-tank workshop was conducted with 14 male and five female GPs, during which the STarT-Back-Screening-Tool (SBST) and related research evidence was presented. This was followed by two focus groups, based on a semi-structured interview guideline to identify potential implementation barriers and opportunities. Discussions were audiotaped, transcribed and coded using a content analysis approach.

**Results:**

For the three deductively developed main themes, 15 subthemes emerged: (1) application of the SBST, with the following subthemes: which health profession should administer it, patients known to the GP practice, the reason for the GP consultation, scoring the tool, the tool format, and the anticipated impact on GP practice; (2) psychologically informed physiotherapy, with subthemes including: provision by a physiotherapist, anticipated impact, the skills of physiotherapists, management of patients with severe psychosocial problems, referral and remuneration; (3) the management of low-risk patients, with subthemes including: concern about the appropriate advising health professional, information and media, length of consultation, and local exercise venues.

**Conclusions:**

The attitudes of GPs towards stratified primary care for LBP indicated positive support for pilot-testing in Germany. However, there were mixed reactions to the ability of German physiotherapists to manage high-risk patients and handle their complex clinical needs. GPs also mentioned practical difficulties in providing extended advice to low-risk patients, which nevertheless could be addressed by involvement of specifically trained medical assistants.

## Introduction

Low back pain (LBP) is among the most common reasons for visiting a general practitioner (GP) both in Germany and other industrialised countries [1, 2]. It is also leading to enormous rising health care expenditure, through increased use of diagnostics and therapy, much of which is not justified by the evidence or by improvements in the quality of treatment provided [3]. Even larger economic consequences result from a loss of work productivity [4, 2, 5].

Numerous biopsychosocial risk factors for persistent disabling LBP have been identified in the literature, [4, 6–8]. Identifying LBP treatments that can effectively target those factors has become the zeitgeist for research and clinical practice over the past 10 to 15 years [9]. Following this focus the STarT Back stratified care approach was developed in the UK (STarT = **S**ubgroups for **Tar**geted **T**reatment) and examined in a randomized controlled trial. Patients in the stratified arm of this trial were categorised into three subgroups based on their prognosis (low, medium and high-risk) using the STarT Back Screening Tool (SBST; available at http://www.keele.ac.uk/media/keeleuniversity/group/startback/Keele_STarT_Back9_item-7.pdf) and provided with matched treatment pathways. All patients received a 30-minute assessment and advice session. Patients that were categorised as low risk were discharged after this one-off session. Patients, who were identified as being at medium risk, received standardised evidence-based physiotherapy addressing symptoms and functioning using treatments like advice, education, exercise and manual therapy that are usual physiotherapy practice in Germany. Patients categorised as high risk were referred for psychologically informed physiotherapy, a treatment which integrates a cognitive-behavioural approach with traditional physiotherapeutic intervention. A detailed description of the treatment protocols and training given to the therapists who delivered the high risk treatments is published [10, 11]. In comparison to the usual physiotherapy control group (non-stratified care), the clinical outcomes for patients randomised to the stratified care approach were significantly better at 4 and 12 months, and cost savings were also evident [10]. In a large-scale follow-up implementation study which embedded the stratified care approach into the routine care delivered by 64 GPs and their associated physiotherapy services, it was demonstrated that stratified care could be feasibly implemented into routine clinical practice and still led to positive clinical outcomes and 50% reductions in patients’ time off work. As a result the wider implementation of stratified care for LBP has been recommended [12]. The study also compliments other evidence suggesting physiotherapists can be upskilled to provide effective enhanced, psychologically informed treatment, for high-risk complex patients [13–16].

In 2012, the SBST was translated and cross-culturally adapted into German, according to internationally accepted guidelines and with approval from the original STarT developers [17, 18]. Due to the regulated practice of physiotherapists, a referral from a medical practitioner is necessary for physiotherapy treatment in Germany, however, the SBST is not currently being used by German GPs in routine care and it is unknown whether this stratified care approach could be implemented into the primary care healthcare system. Organisation of primary care clearly differs between Germany and the UK [19, 20], which may influence how the screening tool is used in practice as well as the appropriate matched treatment pathways [21]. The perspective of the main stakeholders–in the first instance GPs–is of major importance for the implementation of stratified care and has been explored in the UK [22], but not within a German setting. In order to successfully implement stratified care within the German healthcare system, it is clearly important that potential barriers and enablers for this approach are fully explored [23, 24].

Therefore, the primary objective of this study was to explore the views and perceptions of German GPs in respect to using a stratified LBP treatment approach. In particular, we were keen to understand potential organisational and treatment barriers and enablers, in order to identify ideas about how best to adapt the approach to ensure it is practical and meaningful to GPs working in the German healthcare system.

## Methods

A 90-minute think tank workshop was conducted, during which the SBST was introduced and results from the STarT Back trial and implementation study were presented [25, 10]. The presentation was followed by two focus groups. Focus group qualitative methodology was the design of choice for this study because it enables participants to express themselves openly with the added value of social interaction which can stimulate further ideas and conversation.

### Participants and setting

The workshop took place at the University Hospital Heidelberg, Germany. GPs were invited to participate in one of two ways: 1) via an existing GP research network including 86 practices, or 2) via a program for Continuing Medical Education for GP practice teams. All GPs gave their written informed consent before participation. Ethical approval was granted by the Ethics Committee of the University of Heidelberg (registration ID: S-414/2013).

### Procedures

The participants were evenly divided between the two focus groups (9 and 10 participants) which were led by one of two trained facilitators (SK, JSt) using a semi-standardized interview guideline (Table 1). Initially the facilitators invited participants to share their ideas on the presented stratified care approach. Prior to the focus groups the semi-standardized interview questions were tested with a GP who was not a workshop participant [26]. Each facilitator was supported by a research assistant, who took care of organizational aspects of the meeting and took notes during the discussions.

**Table 1:** Interview guideline.

**Introductory question**	How would you use the STarT Back Tool within the therapy planning in your practice?
**Matched treatments**	In your opinion, who should treat high risk, complex patients and in what way?
	In your opinion, who should treat low risk patients and in what way?

The discussions were audiotaped, completely transcribed and coded using a content analysis approach [27]. Transcription was carried out verbatim following predefined department standards by the two research assistants who supported the facilitators and were present during the focus group discussions. The transcripts were re-checked by the two facilitators.

Analysis began by the facilitators becoming immersed in the data through detailed multiple readings of the transcripts (SK, JSt). The main themes and subthemes which emerged from the transcripts were developed by one of the facilitators (SK), organized to align with the order of the interview guideline (SK), and then interrogated by the other (JSt). Through this iterative process the themes were refined collaboratively (SK, JSt). Quotes supporting each identified theme were chosen. As software R-QDA was used for the analysis (2014; http://rqda.r-forge.r-project.org/).

## Results

Altogether 14 male and five female GPs participated, 13 are members of the research network. Six of the participants were 40–49, seven were 50–59, and six were 60–69 years old. On average GPs had been settled with their practice for a mean of 19.1 (±9.5) years (SD). The majority of GPs (n = 8) were from single handed practices, with six GPs from a practice with more than one GP. The participants disclosed very different consultation rates over a yearly quarter, with one GP reporting that they saw between 500–1000 patients, five GPs reported seeing 1001–1500, and five GPs reported seeing more than 1500 patients.

In alignment with the interview guideline, three main themes along with 15 subthemes were established (see Table 2; for quotes see Tables 3 to 5). Two additional main themes were identified inductively from the transcripts: physiotherapy practice, and different types of patients.

**Table 2:** Deductively developed main themes and corresponding subthemes.

Main themes	Subthemes
Applying the screening tool	Administering health professional
	Patients known to the practice
	Reason for the consultation
	Scoring the tool
	Tool format
	Anticipated clinical impact
Psychologically informed therapy	Provision by a physiotherapist
	Anticipated impact
	Skills of physiotherapists
	Severe psychosocial cases
	Referral and remuneration
Management of low risk patients	Advising health professional
	Information and media
	Length of GP consultation
	Local exercise venues

**Table 3:** Applying the screening tool, subthemes and quotes.

Subtheme	Quote
Administering health professional	*Gr2_GP10*: *“Well*, *I think I wouldn’t give this questionnaire to my assistants*, *but rather keep it in a drawer*.*”*
	*Gr2_GP2*: *“After all*, *the patient arrives and checks in at the receptionist and says*: *“I have such terrible back pain” […]*. *Then the [receptionist] hands out this questionnaire*.*”*
Patients known to the practice	*Gr2_GP10*: *“Well*, *during the first consultation I wouldn’t think of doing it*, *except if I knew he gets this problem every three months or twice a year*, *in this case maybe*. *[…]… however*, *never during the first consultation*.*”*
	*Gr1_GP4*: *“One actually knows his patients*, *[…] there is a history of many years*, *[…] in the end it’s twice as much work”*
Reason for the consultation	*Gr1_GP2*: *“In our case the assistants need to ask for the occasion of the consultation*, *this needs to be documented*, *otherwise I won’t accept the patients to my consultation hours*.*”*
	*Gr1_GP4*: *“[…] and those things with the medical assistant*, *that she sorts out things beforehand*. *In practice*, *that’s not possible because people do not always say their diagnosis when standing in front of the desk…” Gr1_GP6 “Lots of them do not even want that*.*” Gr1_GP4 “…yes*, *do not even want that*.*”*
Scoring the tool	*Gr2_GP9*: *“I can create a macro in my PC and the points … –I can write them a line and a questionnaire anyway*, *so and so many points*, *finished*.*” Gr1_GP*?: *“Yes” Gr1_GP9*: *No problem at all*.*”*
	*Gr1_GP5*: *„[…] and I always have concerns regarding time saving*, *because after all I have to evaluate the questionnaire*.*”*
Tool format	*Gr2_GP9*: *"It can also be done with an app*, *[…] with smart phones […]*.*"*
Anticipated clinical impact	*Gr2_GP1*: *“[…] this way I can divide relatively fast into one of three groups and see this or that direction*.*”*
	*Gr2_GP8*: *“[…] the [patients] are astonished themselves what the tool highlights*. *There are also things on it [the tool] they have*, *but are unable to utter verbally*, *don’t describe*.*”*
	*Gr1_GP5*: *“Well*, *I generally have difficulties when using questionnaires because I regard it rather impersonal*, *do not realize the patient’s mimic when asking a respective question*.*”*

**Table 4:** Psychologically informed therapy, subthemes and quotes.

Subtheme	Quote
Provision by a physiotherapist	*Gr2_ GP3*:*”[…] installing this psychosocially trained physiotherapist*, *that would be something very meaningful indeed*.*”*
	*Gr1_GP9*: *“[…]*, *why not actually [treatment by specifically trained physiotherapist]*, *experimentally within a study*.*”*
	*Gr1_GP2*: *“And sometimes I also call the [physiotherapist] and say they actually have a back problem but actually have quite another problem and that must be included in the treatment as well*.*”*
Anticipated impact	*Gr2_GP10*: *“At present it means sending them to a psychiatrist or to a psychotherapy*, *which is a huge barrier and surely not even necessary in the majority of cases*.*”*
	*Gr2_GP3*: *“[…] that [physiotherapist addressing psychosocial aspects] would certainly be a relief for us*!*”*
Skills of physiotherapists	*Gr1_GP5*: *“If I see how much training they undergo in the different courses within their life*, *then they’re enormously qualified*. *In this case*, *only a psychological module would have to be added*.*”*
	*Gr2_GP3*: *“[…]*, *that’s also part of the training today*, *also the entire social component*, *the psychological component*, *or am I wrong here*?*”*
	*Gr1_GP8*: *“Well I think lots of physiotherapists have very good psychotherapeutic accesses to understanding for the patients throughout their longstanding work*.*”*
	*Gr1_GP4*: *“[…] what is really evidence-based in this case–no idea*. *I don’t know either if the physiotherapists do really know that*.*”*
Severe psychosocial cases	*Gr2_GP1*: *“Except it is so dramatic that it cannot be controlled ambulatory anymore*, *he would have to go to a holistic pain clinic or something like that*, *[…]”*
	*Gr2_GP4*: *“[…] but if the symptoms are more severe I think psychotherapy is indicated*.*”*
Referral and remuneration	*Gr1_GP2*: *“[…] I give the patient a referral for physiotherapy subsequently*, *that [need of psychologically informed therapy] is something the therapist only learns by chance*, *and this loss of information*, *that constitutes a problem*.*”*
	*G1_GP4*: *“I think physiotherapy is about 11 or 14 Euros*, *required time is 15 minutes […]*, *and if he is additionally able to achieve an additional service … showing empathy*, *if that contains a conversation*, *the payment should be a lot higher*.*”*

**Table 5:** Management of low risk patients, subthemes and quotes.

Subtheme	Quote
Advising health professional	*Gr2_GP8*: *“Yes*, *I’ve got that in hand*, *I advise them*, *I want to see the patient every week*, *I want to see progress*, *what is being done*.*”*
	*Gr2_GP1*: *“Either it is a member of the consultation staff that takes over yet another part*. *[…] Or you say*: *‘Well*, *physiotherapist XY is particularly trained for this*, *in that case as in yours and is able to advise you*. *Tomorrow you’ll have an appointment*.*’”*
Information and media	*Gr1_GP9*: *“Well*, *I could well imagine that patients rather look at images*, *moving images than reading a text and finally really adopt it*.*”*
	*Gr1_GP7*: *“I believe videos somehow fizzle out*.*”*
	*Gr2_GP10*: *“Another possibility would be to sit him in the waiting room or at a free PC computer point for watching the video*.*”*
	*Gr2_GP10 “Can you give them the video to watch it at home*?*”*
Length of GP consultation	*Gr2_GP10*: *“Well*, *let’s put it this way*, *30 minutes are not impossible in everyday life*. *The question is what can you take from it and either give it to a non-medical staff or to a physiotherapist*.*”*
	*Gr2_GP9*: *“Half an hour would never ever be possible for us at the moment*.*”*
Local exercise venues	*Gr1_GP6*: *“[…] many offers we have to search must also be searched by the neighbouring practice in the same way instead of publishing it on a platform*.*”*
	*Gr1_GP7*: *“[…] the [patient] wants a qualitative evaluation from us*.*”*

### Applying the screening tool

Aspects which were discussed by the GPs in respect to implementing the SBST were: the most appropriate administering health professional, if the patient was already well known to the practice, the enquiry of the reason for the consultation, tool scoring, tool format, and its anticipated clinical impact.

In respect to organisational aspects GPs discussed if they themselves or their medical assistants would be most appropriate to administer the screening tool. Some GPs favoured delegating the task, whilst others were against this suggestion.

During the discussion it became clear that individual GP practices are organised very differently in respect to how much information GPs know beforehand about the main reason for the consultation. In some practices medical assistants routinely ask patients about the reason for their visit, whilst for others this was not considered appropriate.

The issue about patients who were known to the practice as having recurrent LBP problems also led to differences in opinion among different GPs regarding how best to administer the tool. Some GPs felt it was inappropriate to ask patients to complete the SBST during their first consultation for LBP, whereas others felt this would be a possibility.

Scoring the tool was discussed in context with time resources and the tool’s format. Individual participants reasoned that using the tool would require additional resources, as calculating the STarT-score and determining the patient’s risk-group might take time. Others opposed, suggesting that the instrument with only nine items was sufficiently short to be practical for use within short consultations. In order to improve implementation, some GPs suggested embedding the tool within the GP computer or having it available on a tablet or mobile device. If this was possible, then calculating the score and risk-group could be automatic and so save the GPs time.

The impact the tool might have on GP decision making was an important discussion point. Some physicians thought the tool was likely to be a useful way of assisting their treatment plans for an individual. GPs tended to view the tool as a potential resource that might help to speed up their clinical decision making process, and that it could support their explanations to patients about their likely prognosis and how best to manage their condition. Other GPs in contrast did not expect it to be of much assistance to their decision making and some felt it might have a negative influence on the consultation because it was impersonal and involved disturbing non-verbal communication methods between the patient and their doctor.

### Psychologically informed therapy

In respect to implementing psychologically informed physiotherapy, the majority of GP comments were focussed on the physiotherapists as the professionals to deliver this care. Implementation of an effective treatment approach for complex high-risk patients was anticipated as beneficial. The GPs wished for support for treatment of these patients. In this context, the skills and qualifications of physiotherapists were discussed intensively, including their abilities to identify patients that might need formal psychological therapy with a specialist clinician. In addition, the need for some adaptation of physiotherapists’ current working conditions was discussed in order to ensure that more time was available for these patients.

A further controversial discussion point was the issue of implementing physiotherapists who deliver psychologically informed physiotherapy, especially since the participants agreed that treating high risk, complex patients could be very clinically challenging. Therefore, several GPs expressed the view that training physiotherapists to better manage psychosocial aspects among high-risk patients might be particularly beneficial. One GP reported in fairly isolated cases to already asking the physiotherapist to specifically consider psychosocial obstacles.

Overall, GP participants were open minded about the stratified care approach to LBP, but recommended that implementation should be coincided with a robust scientific evaluation of the impact of this new approach in the German system.

Another theme that was comprehensively discussed was whether physiotherapists needed formal educational skills and qualifications or simply could be expected to learn and gain experience by doing. GPs felt that physiotherapists had appropriate skills to manage complex biomedical needs and expected that psychological competencies were something which came to some extend with the right experience and so could be within their scope of practice. Potential limitations of even specifically trained physiotherapists, to address the needs of some patients with severe complex psychological problems were discussed (i.e. needing treatment by a psychologist or in a specialized pain clinic).

Uncertainties were however expressed, about the dimension to which psychosocial competencies were currently included within physiotherapy training programmes, and they also were uncertain about how knowledgeable physiotherapists were about evidence-based practice.

In respect to physiotherapists’ working arrangements and appointment times, there was broad recognition by the GPs for the need for some system modifications. The participants reflected on the current practice of communication between GPs and physiotherapist which, in nearly all cases is solely based on the referral form. For effectively implementing the STarT-approach, the flow of information would have to be improved. Additionally, the remuneration for the treatment of high-risk complex patients was a point of discussion. This was mentioned as a task of high value which should be acknowledged.

### Management of low risk patients

The following themes emerged from the discussion about implementing the matched treatment for low-risk patients; the most appropriate professional to advise patients, the length of consultations, media that should be used to support information given, and information about local exercise venues. The alternative health professionals that were highlighted and considered in this discussion were GPs, physiotherapists, and medical assistants. In Germany, it is current practice that LBP patients receive advice and education from their GP, but some GPs were open to this task being shared with physiotherapists, on the condition that this type of appointment would be available within a very short waiting time.

In order for low-risk patients to receive their advice and support for self-management the issue of the spatial and organisational distance between physiotherapists and GPs was seen as a barrier, especially due to the need for patients to receive immediate advice. The medical assistant in the GP practice was seen as primarily an administrative role which could be useful.

Concerning the use of advice booklets, GPs had mixed experiences and the use of an informational video to help educate patients was rare. Their expectations about the success of such an approach varied. They also highlighted that providing patients with an advice/educational video would probably require additional structures which were currently not available within their practices, although, as a possible solution some suggested to put a computer in the waiting room. Alternatively different strategies like online videos or DVDs for patients to view at home were proposed.

The duration of the initial 30-minute assessment and advice appointment for patients in the STarT approach was identified as a potential barrier for GPs due to perceived time constraints under the current working conditions even though it did not seem impossible, especially if supported by the medical assistant.

Some GPs felt comfortable that they knew about local opportunities for patients to exercise, although others did not feel they had a comprehensive overview of such venues and were open to having support to collate this information. It was felt that it was important for GPs to be able to recommend local facilities if patients were to have confidence in them, although most GPs did not feel prepared to provide such an evaluation for their patients. Concerns were put forward regarding potential costs, although health insurance financed options were discussed as an alternative.

### Inductively identified themes

In the analysis two additional themes with relevance to the implementation of the STarT Back approach in Germany were identified inductively: GPs views about physiotherapy and GPs perceptions about different types of patients.

For physiotherapy it was discussed that there is a lack of information about practice: *“Well*, *that would be desirable*, *if you knew what the physiotherapist really does in this case” (Gr2_GP1)*. In Germany, due to the regulated practice of physiotherapists, a referral from a physician is necessary. The regulation process is complex and many physicians in ambulatory care fear a financial penalty as part of their liability as a result of over-referring. During the discussions it was stated, that physiotherapists should share financial responsibility: *“Yes*, *the [physiotherapists] must sit in the budget-boat*.*” (Gr2_ HA8)*.

The GPs also felt that there was an important patient-type that was not specifically identified or given appropriate consideration in the STarT Back study. With *“Rentenbegehren”(Gr1_GP1)* patients are meant who have a strong desire to retire early and to get a pension due to their low back pain (Rente = pension, Begehren = desire). This specific German term was repeatedly mentioned.

## Discussion

Evidence based treatment approaches are often implemented into clinical practice in an ad hoc fashion [28]. The presented study aimed to explore the views and perceptions of German GPs in respect to using and implementing a stratified LBP treatment approach in the German healthcare system.

Concerning the stratified care approach, overall, GPs were positive. This became particularly clear in relation to identifying and treating the small but demanding group of high-risk patients and would clearly be important aspect to get right in achieving successful implementation in Germany [22]. The GP participants therefore agreed that there is a prevalent subgroup of LBP patients who need psychosocial informed treatment, but not to the extent that a referral to a psychologist would be appropriate. In fact, GPs expressed reservations about referring LBP patients to a psychologist because this was usually perceived as a much bigger hurdle in comparison to being referred to a physiotherapist. As a result GPs felt that training of physiotherapists to better address the needs of complex, high risk patients would be beneficial to the German healthcare system.

Although the importance of fast tracking high-risk patients was seen, participating GPs stated that they would prefer not use the screening tool during the first consultation of a patient. Others feared that the use of the tool might be impersonal and have a negative influence on the communication with the patient, but also positive effects like the use of the validated instrument to stratify patients in relation to their treatment prognosis or the possibility to use the tool as a starting point for the subject history taking were anticipated.

Within the STarT Back approach information leaflets and videos as well as advice about the details of local exercise venues are recommended to help support self-management [10]. During the discussions it became evident that currently not all GPs are used to providing leaflets about back pain and there were participants who were sceptical about using an educational video as part of their treatment. In addition, several GPs did not feel they had the resources to gather information on local exercise venues and their quality.

Various contextual factors related to the practice organisational structures were identified. In respect to administering the screening tool, GPs had different opinions about the option of using medical assistants to do this task, particularly as in many practices patients are not asked for the reason for their consultation before they are seen by the GP (for a description of the competencies of "medical assistants" see [20]). In relation to finding time to give education and advice to patients, there was a general feeling that GP consultations are limited because of time constraints. The majority of the participants reported to see more than 1000 patients quarterly. This fits to the mean consultation time of 9 minutes described for German GPs [29]. This is very short, particularly when considering that there are high need patients like those with a high psychosocial burden or the desire for a workers' compensation. In addition, in some practices the spatial constraints would be a barrier to patients being shown an educational video.

Other contextual factors were discussed. In general, the idea of specifically trained physiotherapists to treat high risk, complex patient was very positively received. Physiotherapists were described as being particularly skilled in managing biomedical, physical conditions. In contrast, GPs were uncertain about physiotherapists’ skills in managing patients with complex biopsychosocial problems and felt that appropriate training would be important to prepare them for this extended role. Another theme which emerged was the importance of inter-professional collaboration, particularly the wish to improving the flow of information between the healthcare providers.

Another important contextual factor addressed by the participants was financial pressures [30]. A facilitator for a stratified care approach in Germany would be given, if implementation would reduce the probability of a financial recourse like it is currently feared by German GPs.

### Implications for practice organisation

Overall, participants agreed that the SBST’s brevity and ease of scoring make it feasible and practical for use in general practice [25]. However, they suggested that an electronic version of the tool with automated scoring would be a further facilitator for its implementation. This type of advancement has been undertaken in the UK [31] and is possible within German primary care. Moreover, a free app is available for mobile phones and could be translated into German [32].

Time constraints appear to be a perceived barrier to GPs feeling able to advise LBP patients during their consultation. However, the alternative of implementing physiotherapists to perform this task was considered to be impractical due to the geographical and organisational separation between physicians and physiotherapists. One proposed solution is for GPs to provide patients with some brief advice that is then followed up through a further session with a physiotherapist. This model would be similar to the procedure described by Sowden et al. for the IMPaCT Back implementation study. In this project patients who were classified as medium or high risk by the GP were sent to a physiotherapist, although those who improved and were classified as low risk after re-assessment at the physiotherapy practice, were only provided with a one-off assessment and advice session [33].

One alternative suggestion to increase the time available to give advice to patients within the GP practice was to involve GP medical assistants. In Germany such medical assistants are mainly administrative roles, but there are some attempts to broaden their competencies [20]. For example, it was felt appropriate that medical assistants could provide information about local exercise venues, written education material and to show patients an educational video. A possible strategy to overcome the limitations of physical space within the practice to deliver video material was suggestions to offer web-based material that patients could watch at home or on their mobile device. One notable finding was that not all GPs were aware of available informational material for LBP patients, and so ensuring such information was easily accessible would be an important component for a stratified care implementation program.

### Training for GPs

The positive perceptions of the GPs concerning the STarT Back stratified care approach is a potential enabling factor for future GP training and implementation. GPs should be informed about the evidence on biopsychosocial treatment effects e.g. the impact on clinical outcomes [34, 35], but also need practical details on how this approach might influence patient care and clinical pathways both for themselves, and for related physiotherapy services and their ability to treat particularly the high risk complex patients group [10, 36, 15]. This also applies for video-based information [37, 10]. An important patient-group that might need more consideration in the STarT Back approach are those with a strong desire to retire early [38]. A specific training to help to manage these patients could be of benefit.

One important finding was that GPs could have difficulties with the idea of changing from existing stepped care models (waiting to see how patients symptoms develop) to the stratified care model (acting decisively based on patient’s initial SBST prognostic profile). As a result, this aspect should be addressed intensively within a training program.

Another change for GPs was the use of prognostic information during a LBP consultation, and it was apparent that here a training need also existed as GPs had the perception that use of such a tool during the GP consultation would make the patient interaction feel more impersonal. Accordingly, it would be relevant to explain that the items contained in the SBST may help patients to relate more honestly about the impact their pain is having on them, particularly in respect to their mood.

To address the concerns GPs raised about financial aspects it could be important to investigate the cost effectiveness of the STarT Back stratified care approach during its implementation into the German healthcare system. The UK implementation has demonstrated the approach to be broadly cost-neutral with extra resources used for relatively small group high-risk patients being made available from savings gained in the treatment of the large group of low-risk patients [12, 10].

### Strength and weaknesses

The varying topics discussed in the focus groups highlighted that the GPs were able to grasp the STarT Back approach despite a relatively short introduction. Unclear points were readily clarified during discussions. Accordingly, the transcripts could be evaluated effectively according to the initial aims.

The results convey the impression that with regard to the described aim, a satisfying saturation was reached. For qualitative group approaches, Carlsen and Glenton identified recommendations ranging from two to five groups adding that the necessary number depends on the research question and the composition of the groups [39]. Recommendations concerning the number of participants range between 6 and 12 [40]. We are of the opinion that with two groups of altogether 19 participants, we received a saturation fitting our research purpose, which was satisfactory although the duration of the discussions was short. Reasons for reaching our goals with this compact design relate to preparing a clear introduction and discussion guideline.

### Research agenda

Research on the psychometric properties of the German version of the SBST ran in parallel to the described work. Foster et al. propagate randomized trials as a preferred design for testing stratified care [9]. Correspondingly, the next step should be a pilot study examining the adapted approach and also providing information on the developed implementation strategy.

Patient expectations are believed to play an important role on treatment outcome [41]. The physicians participating in our study mentioned several ideas on how patients would react to different aspects of the STarT-approach if implemented in Germany. Those should be discussed with patients and additional aspects should be gathered.

Similarly, this applies to physiotherapists. Questions need to be discussed, e.g. their attitudes towards evidence-based practice, their qualifications and ability to address psychosocial factors or concerning the one-off clinic appointment, as described for the STarT-approach [10].

## Conclusions

The positive attitudes of GPs towards stratified primary care for low back pain patients indicate good potential for pilot-testing the STarT approach in Germany. The implementation might be an opportunity to enhance collaboration between GPs and physiotherapists. To prepare a receptive environment for this, the ability of trained German physiotherapists to address psychosocial risk factors needs to be determined and communicated. A carefully planned initiation of the approach for low risk patients is important, since this treatment arm presents a clear contrast to current practice. Because of the short consultation time for GPs in Germany it is unrealistic that GPs alone could carry out the advice process as it originally exists in the STarT Back Trial. Support by specifically trained staff in the GP practice might be a possibility.

Further qualitative research with physiotherapists and patients to evaluate how to implement stratified care for LBP patients within Germany are underway. The combined results should lead to a randomized pilot trial to test the adapted treatment approach.
